# DNMT1 as an environmental sensor: epigenetic pathways linking environmental exposures, sex hormone signaling, and vulnerability to neurodevelopmental and neurodegenerative diseases

**DOI:** 10.3389/fneur.2026.1883887

**Published:** 2026-07-29

**Authors:** Katharina Vöhringer, Magdalena Sophie Müller, Can Bora Yildiz, Geraldine Zimmer-Bensch

**Affiliations:** 1 Division of Neuroepigenetics, Institute of Zoology (Biology 2), RWTH Aachen University, Aachen, Germany; 2 Research Training Group 2416 MultiSenses – MultiScales, RWTH Aachen University, Aachen, Germany

**Keywords:** DNA methylation, DNMT1, environmental epigenetics, microbiome-brain axis, neurological disorders, sex differences

## Abstract

DNA methyltransferase 1 (DNMT1) has classically been viewed as the canonical maintenance methyltransferase, yet accumulating evidence positions it as a multifaceted hub that integrates environmental, hormonal, and metabolic signals with chromatin regulation in the developing and adult brain. This review highlights DNMT1 as an environmentally responsive epigenetic sensor across the lifespan. We examine how psychosocial stress, early-life adversity, inflammation, nutritional and microbiome-derived metabolites, and environmental toxicants modulate DNMT1 expression, subcellular localization, and post-translational modifications, thereby reshaping DNA methylation landscapes in neurons and glia. We further discuss how sex hormone signaling, particularly estrogen receptor alpha *α* (ERα)-DNMT1 feedback loops, introduces sex-specific dimensions to epigenetic responsiveness, and how lncRNAs serve as intermediaries linking environmental cues to targeted DNMT1 recruitment at specific genomic loci. Building on this framework, we review how DNMT1 dysregulation contributes to neurodevelopmental and neuropsychiatric disorders—including schizophrenia, autism spectrum disorder, and depression—and to neurodegenerative conditions such as Parkinson’s disease, Alzheimer’s disease, amyotrophic lateral sclerosis, and DNMT1-associated monogenic neurodegenerative disorders. By positioning DNMT1 as a molecular interface between genetic predisposition, environmental exposure, and circuit-level vulnerability, this review highlights the need for integrated, sex-stratified, and longitudinal approaches to understanding epigenetic risk in neurological disease.

## 1 Introduction

The developing and adult brain relies on a dynamic interplay between intrinsic genetic programs and extrinsic environmental signals to build and maintain complex neural circuits (1–3). Epigenetic mechanisms provide a molecular interface for this dialogue by translating developmental cues, neuronal activity, and environmental exposures into stable yet plastic changes in gene expression (4, 5). Among these mechanisms, DNA methylation by DNA methyltransferases (DNMTs) plays a pivotal role in neuronal differentiation, synaptic function, and experience-dependent remodeling (6–10). The three major DNMTs—DNMT1, DNMT3A, and DNMT3B—have distinct but partially overlapping functions, with DNMT3A and DNMT3B acting primarily as *de novo* methyltransferases, and DNMT1 long regarded as the canonical maintenance enzyme that preserves methylation patterns during DNA replication (10).

Over the past decade, the traditional view of DNMT1 as merely a maintenance methyltransferase has been expanded. Emerging evidence indicates that DNMT1 participates in the integration of diverse environmental and cellular signals, including psychosocial stress, metabolic pathways, environmental toxicants, sex hormone signaling, and long non-coding RNAs that modulate its genomic targeting and subcellular localization (11–15). Converging evidence from human genetics and postmortem studies further links DNMT1 dysregulation to a spectrum of neurodevelopmental, neuropsychiatric, and neurodegenerative conditions (16–23).

In this review, we propose that DNMT1 can be viewed as an environmentally responsive epigenetic hub. After providing a brief overview of DNMT1’s molecular identity and canonical brain properties, we examine how stress, nutrition, microbiome-derived metabolites, environmental toxicants, lncRNAs, and post-translational modifications tune DNMT1 activity. We next discuss how sex hormones introduce sex-specific dimensions to this regulation. Building on this framework, we review how DNMT1 dysregulation shapes vulnerability to neurodevelopmental, neuropsychiatric, and neurodegenerative diseases, and discuss future directions and translational potential.

## 2 DNMT1: molecular identity and canonical functions in the brain

DNMT1 is the primary maintenance DNA methyltransferase, responsible for copying hemimethylated CpG dinucleotides generated during DNA replication to the newly synthesized strand, thereby preserving cell type-specific methylation patterns across cell divisions (10). This function is essential during early brain development, where DNMT1 is highly expressed in proliferating neural progenitor cells (NPCs) and ensures the faithful transmission of lineage-specific epigenetic programs (24–26). Beyond this classical role, DNMT1 remains expressed in postmitotic neurons and glia throughout adulthood, where it participates in activity-dependent DNA methylation, synaptic plasticity, and circuit maintenance (7, 27–30). Molecular dynamic simulations, as well as biochemical and genomic studies have further shown that DNMT1 can bind to unmethylated DNA and catalyze *de novo* methylation at selected regulatory regions, engaging also in non-canonical functions through a crosstalk with histone-modifying complexes such as PRC2/EZH2, and even extranuclear interactions with cytosolic proteins impacting cytoskeleton remodeling (10, 31–35). These diverse functions position DNMT1 as a pleiotropic regulator of neural circuit integrity whose activity extends to environmentally modulated epigenetic programming across the lifespan.

## 3 DNMT1 as an environmentally modulated hub

The brain develops and functions in continuous interaction with its environment, and epigenetic mechanisms are increasingly recognized as central mediators of this dialogue (11). DNMT1 is particularly well positioned to integrate environmental information as it operates at the intersection of developmental programming, neuronal activity-dependent plasticity, metabolic status, and cellular stress responses across the lifespan (10). Environmental signals can alter DNMT1 expression, subcellular localization, post-translational modification, and access to methyl-group donors, thereby reshaping both DNA methylation landscapes and non-canonical DNMT1 functions in neurons and glia (20, 36–38). By tuning DNMT1 activity, exposures such as psychosocial stress, inflammation, and microbiome-derived metabolites can influence neuronal development and identity, synaptic function, and long-term circuit stability, ultimately modulating vulnerability to neurodevelopmental and neurodegenerative disorders (11, 39, 40).

### 3.1 Stress, early-life adversity, inflammation, and brain DNA methylation

Psychosocial stress and early-life adversity are prominent environmental risk factors for neurodevelopmental and neuropsychiatric disorders, and multiple human and animal studies implicate altered DNA methylation in stress-responsive genes as a mechanistic link (11). Acute and chronic stress can induce genome-wide shifts in DNA methylation in brain regions involved in emotion and cognition and are associated with differential expression of DNA methyltransferases, including DNMT1 (41, 42). Deletion of *Dnmt1* in neural stem cells during prenatal development not only causes impaired neurogenesis but also triggers neuroinflammatory changes, such as increased GFAP expression and microglial activation, underscoring the tight coupling between DNMT1 function, stress/inflammatory signaling, and brain homeostasis (43). Inflammatory microenvironments in the brain, for example after radiotherapy in the central nervous system (CNS) or in the context of metastatic brain tumors, are associated with cell-type-specific DNA methylation changes that may be mediated in part by altered DNMT1 activity in microglia, astrocytes, and neurons (44, 45). However, it is important to emphasize that while stress and inflammation broadly alter the brain’s methylome, direct causal evidence establishing DNMT1 as the exclusive or primary mediator of these changes in the CNS remains limited. In many contexts, DNMT1 alterations may be secondary to broader epigenetic perturbations and inflammatory responses, representing a critical gap for future investigation.

### 3.2 Metabolic, nutritional, and microbiome-derived modulation of DNMT1

DNMT1 catalytic activity depends on the availability of methyl-group donors and cofactors, such as S-adenosylmethionine (SAM), generated by folate and methionine cycles, making it intrinsically sensitive to nutritional and metabolic status (46). Dietary patterns that alter one-carbon metabolism, as well as systemic metabolic disorders, can impact DNMT1-dependent methylation capacity and thereby influence gene expression programs in developing and adult brain cells (47). The gut microbiome adds an additional layer to this axis: microbiome-derived metabolites, including short-chain fatty acids, can modulate DNA methylation, histone acetylation, and noncoding RNA expression in the brain and are increasingly recognized as potential modulators of the neuroepigenome (40, 48). Conceptually, microbiome- and diet-dependent changes in inflammation, energy metabolism, and metabolite availability are highly likely to shape the epigenetic milieu in which DNMT1 operates. Nevertheless, precise DNMT1-centered mechanisms in the CNS remain to be fully elucidated, as current models are mostly inferred from broader metabolic studies or peripheral tissue rather than direct experimental demonstrations in neural systems.

### 3.3 Environmental toxicants, air pollution, and lifestyle-related exposures

A broad spectrum of environmental toxicants, including heavy metals, pesticides, and air pollutants, has been linked to altered DNA methylation profiles and increased risk for neurodevelopmental and neurodegenerative disorders (12). For example, traffic-related fine particulate matter (PM2.5) exposure is associated with differential DNA methylation at loci involved in inflammation and Alzheimer’s disease neuropathology in human brain tissue, supporting a role for environmentally induced epigenetic reprogramming in neurodegeneration (49). Many toxicants converge on oxidative stress, mitochondrial dysfunction, and inflammasome activation, cellular states in which DNMT1’s nuclear and extranuclear functions are strongly engaged (50–52). Oxidative DNA damage and redox imbalance can interfere with DNMT1 binding and methylation fidelity, while stress-activated kinases and DNA damage sensors such as ATM can modulate DNMT1 post-translational modifications (PTMs), stability, and recruitment to damaged chromatin (53). Lifestyle-related exposures such as chronic alcohol consumption or sleep disruption similarly influence oxidative stress and metabolic homeostasis, providing additional entry points by which environmental factors may perturb epigenetic states (54, 55). Although a biologically plausible connection could be established linking DNMT1 function to stress and metabolic factors, distinguishing direct toxicant-induced functional changes of DNMT1 from secondary DNA methylation adjustments remains a major challenge. Consequently, the specific contribution of DNMT1 to toxicant-induced neurotoxicity should currently be viewed as an emerging hypothesis warranting targeted investigation.

### 3.4 LncRNAs as mediators of DNMT1-based epigenetic regulation

Long non-coding RNAs (lncRNAs) have emerged as versatile regulators of chromatin architecture, transcription, and RNA processing and are increasingly recognized as key mediators of environmental influences on gene regulation (56). Many lncRNAs display cell type-specific and stimulus-responsive expression and can act as scaffolds, guides, or decoys for chromatin-modifying complexes, thereby linking extracellular cues such as hormones, stress, nutrients, and inflammatory signals to local chromatin states. In plants, non-coding RNAs have been shown to mediate environmentally induced DNA methylation changes in response to abiotic stressors such as temperature, salinity, and nutrient deprivation (57, 58).

In mammalian cells investigated in cancer and peripheral tissue models, DNMT1-associated lncRNAs have been identified that physically interact with DNMT1 and regulate its genomic occupancy and downstream methylation patterns (13, 59–61). For instance, the lncRNA *DACOR1* binds DNMT1, promotes changes in DNA methylation and gene expression in colon cancer cells without altering total DNMT1 protein levels. The original study also reported repression of cystathionine *β*-synthase, which suggests a possible indirect effect on methionine and SAM metabolism, and thus a feedback onto DNA methylation capacity, although this was not directly demonstrated as a mechanistic pathway (60, 62). In a cell model for medulloblastoma, the lncRNA *Snhg15* has been shown to recruit DNMT1 to the *Ncam1* promoter by forming an RNA/DNA triplex, inducing targeted methylation and silencing (13). Interestingly, the expression of *Snhg15* was diminished when cells were stimulated with recombinant ephrinA5, a signal known to regulate interneuron migration and modulate axonal pathfinding (63, 64). This work provides a mechanistic example of how an extracellular guidance cue can reprogram cell behavior by transcriptionally regulating a lncRNA that, in turn, targets DNMT1 to specific genomic sites via triplex formation. Beyond locus-specific recruitment, lncRNAs can also govern DNMT1 subcellular localization: nuclear retention of DNMT1 has been shown to depend on lncRNA interaction, with depletion of such factors resulting in cytosolic redistribution of DNMT1, widespread DNA hypomethylation, and genomic instability (65). While many of these direct mechanisms have been established in non-neuronal systems, they provide a strong conceptual framework for how environmental and microenvironmental signals might shape neuronal gene expression and disease-associated transcriptional reprogramming through locus-specific DNA methylation changes.

### 3.5 Environmental regulation of post-translational modifications of DNMT1 and other epigenetic modifiers

Beyond transcriptional control and changes in metabolite availability, environmental factors can influence epigenetic regulation by modulating PTMs of DNMT1 and other chromatin modifiers. DNMT1 is subject to multiple PTMs, including phosphorylation, acetylation, ubiquitination, and sumoylation, that collectively determine its stability, subcellular localization, interaction with partner proteins, and enzymatic activity (66). Phosphorylation at Ser154 by cyclin-dependent kinases alters domain interactions and impacts both activity and protein stability, and dysregulated CDK signaling under pathological or stress conditions can lead to aberrant DNMT1 phosphorylation and DNA hypermethylation (67). DNA damage and oxidative stress activate ATM, which coordinates DNMT1 acetylation by the histone acetyltransferase Tip60 and subsequent ubiquitination via UHRF1, thereby coupling genotoxic stress to DNMT1 degradation and changes in methylation maintenance (68). Importantly, this axis is also responsive to neuronal activity: in cultured hippocampal neurons, activity-dependent signaling promotes Tip60 nuclear import alongside remodeling of neuroplasticity gene loci, indicating that synaptic activity itself feeds into the ATM-Tip60 pathway and can influence DNMT1 turnover and methylation fidelity in the brain (69).

Similar environment-sensitive PTM-based regulation governs chromatin modifiers that are functionally linked to DNMT1. HDACs and EZH2, which physically associate with DNMT1 in transcriptional repressor complexes, exhibit altered activity and PTM states in response to stimuli (34, 70–76). Given their functionally opposing roles in chromatin regulation, it is notable that histone acetyltransferases such as CBP/p300 and GCN5 are similarly responsive to environmental stressors. For instance, metabolic shifts directly tune GCN5 catalytic activity via substrate availability, while acute oxidative stress induces rapid auto-acetylation and stabilization of p300 (77, 78).

While many of these functional interactions have primarily been characterized outside the nervous system, environmentally induced shifts in these co-regulators could plausibly alter the composition, stability, and target recruitment of DNMT1-containing complexes in the brain. Such PTM-driven crosstalk provides a compelling conceptual framework for how metabolic and stress signals might eventually lead to disease-related epigenetic dysregulation in neurons. Together, these converging pathways suggest that DNMT1 operates within a broad, environmentally sensitive regulatory network, though the precise neural mechanisms remain to be fully established. Ultimately, this local epigenetic regulation is further shaped by systemic endocrine factors, which provide an additional long-range layer of control over DNA methylation dynamics.

## 4 Sex hormones as modulators of DNMT1 and DNA methylation

### 4.1 DNMT1 as a key player in hormone-dependent DNA methylation

Sex hormones are steroid hormones that regulate the development and maintenance of sex-specific characteristics and reproductive functions. Although present in both sexes, they differ in circulating levels and physiological effects, and can regulate epigenetic mechanisms directly or indirectly through binding to nuclear hormone receptors (79). In particular, the expression of DNMT1, DNMT3A and DNMT3B has been shown to be modulated by sex hormones (14, 80, 81). Bioinformatic analyses have identified multiple binding sites for estrogen receptor alpha (ERα) within the promoter regions of these DNMT genes (82). Consistent with these predictions, ERα has been demonstrated to directly bind to the promoters of *Dnmt1* and *Dnmt3a* in human breast cancer cells, with selective modulation of DNMT1 expression altering global DNA methylation levels and a positive correlation between ERα and DNMT1 expression (83). In the mouse cortex, DNA methylation suppresses ERα expression, suggesting a feedback loop that contributes to the regulation of the ERα-DNMT balance (84). Notably, methylation of the *Esr1* gene promoter in the cortex is age dependent, with ERα expression declining after postnatal development, accompanied by reduced DNMT3A and increased DNMT1 mRNA levels. Furthermore, ERα expression in the hypothalamus fluctuates across the estrous cycle of female mice, with high expression levels during proestrus compared with diestrus and after ovariectomy, suggesting that hormonal cues dynamically regulate the ERα-DNMT feedback loop (85).

Taken together, DNA methylation mediated by DNMT1 is closely associated with ERα expression, suggesting a potential sex-dependent role of epigenetic mechanisms. However, the causal relationship remains incompletely resolved, as it is not yet clear whether changes in DNA methylation drive alterations in sex hormone signaling or whether this signaling induces modifications in DNA methylation. The relationship is likely bidirectional, consistent with the proposed regulatory feedback loop between DNA methylation and estrogen signaling.

### 4.2 Neurobiological consequences of sex-dependent epigenetic regulation

Sex differences in DNA methylation have been reported across multiple brain regions, suggesting sex-specific epigenetic regulation of gene expression in the brain (86). These differences have been suggested to contribute to sex-specific vulnerability to neurological and psychiatric disorders (87). Regulatory networks derived from sex-differentially methylated genes in human post-mortem brains show significant enrichment for genes associated with psychiatric disorders (88). This suggests that sex-dependent DNA methylation contributes to the molecular mechanisms underlying sex biases in disease susceptibility. However, these observations are largely correlative and reflect sex-dependent epigenetic associations rather than established DNMT1-driven mechanisms.

Fluctuations in ovarian hormone levels are thought to contribute to heightened risk for mood and anxiety disorders in women, highlighting a hormonal mechanism that may reinforce sex-specific vulnerability (89, 90). This is exemplified by the estrous cycle-dependent transcription factor Egr1. When overexpressed in the ventral hippocampus, Egr1 directs neuronal chromatin opening in both sexes and is regulated by estrogen via estrogen receptors (91). Notably, Egr1 expression positively correlates with estrogen levels and negatively with anxiety-related behaviors, suggesting a direct link between sex hormones, chromatin regulation and behavior. Extending these mechanistic insights to the systems level, single-cell profiling of the female mouse brain across reproductive transitions reveals widespread, hormone-sensitive remodeling of brain plasticity, particularly in excitatory neurons and neural stem cell populations (92). In the human cortex, convergent cell type-resolved analyses reveal that sex-biased gene expression is highly cell type-specific, particularly in glial populations and excitatory neurons (93). Collectively, these findings support a multi-scale role for sex-dependent epigenetic regulation in shaping brain cell type-specific plasticity and disease vulnerability. These observations further suggest that DNA methylation represents a key epigenetic layer associated with sex differences in disease susceptibility, highlighting the need for further research to disentangle causal mechanisms and identify the specific contribution of enzymes such as DNMT1.

### 4.3 Environmental modulation of sex-specific epigenetic mechanisms

Sex differences in epigenetic mechanisms are not only regulated by sex chromosomes and gonadal hormones, but also by environmental factors. Several studies in peripheral tissues have reported sex-specific epigenetic responses to environmental toxicants: Childhood lead exposure and prenatal exposure to endocrine disruptors, including BPA and MCPP, produce divergent DNA methylation outcomes in males and females (94, 95). This sex-dependent epigenetic response is also evident at the hormonally relevant locus *ESR1*, for which MCPP-associated methylation differences have been reported in infant cord blood (94). However, direct evidence for analogous DNMT1-dependent mechanisms in the CNS remains limited. Given the established ERα-DNMT1 feedback loop and the sensitivity of lncRNA expression to both hormonal and environmental cues, analogous sex-stratified responses are conceivable in the brain. However, the precise role of DNMT1 in mediating such effects in the CNS remains insufficiently understood and represents an important research gap for clarifying how epigenetic regulation contributes to sex differences in response to environmental exposures and in the pathophysiology of neurodevelopmental and neurodegenerative disorders. Together, these observations highlight a biologically plausible but largely indirect set of connections between environmental exposures, epigenetic regulation, and sex differences in brain function, underscoring the importance of an integrated perspective. In this context, DNMT1-dependent DNA methylation may represent a regulatory interface linking hormonal and environmental influences to sex-specific neural outcomes, although direct mechanistic evidence in the CNS remains limited. This is particularly relevant in light of reported associations between DNMT1-related epigenetic regulation and neurodevelopmental and neurodegenerative disorders.

## 5 DNMT1 in neurodevelopmental and neuropsychiatric disorders

### 5.1 DNMT1 as a link between epigenetic regulation, neurodevelopment, and disease vulnerability

DNMT1 is a key maintenance DNA methyltransferase that helps preserve DNA methylation patterns during cell division and thereby supports the stabilization of cell identity during neurodevelopment (19, 96). In the developing nervous system, DNMT1 remains expressed beyond proliferating progenitor stages and contributes to postmitotic neurodevelopmental processes. In cortical interneurons, DNMT1 regulates migratory behavior and timing of cortical plate invasion, supports migration-associated cellular morphology and neuronal survival, and contributes to the regulation of transcriptional programs linked to interneuron maturation and subtype specification during cortical development (21, 97, 98). However, DNMT1 acts within a broader epigenetic framework in which genetic predisposition and environmental influences jointly shape neurodevelopmental trajectories (96). During sensitive developmental periods, dynamic DNA methylation contributes to neuronal lineage decisions, maturation, and circuit assembly, and disruption of these tightly regulated processes can leave persistent changes in DNA methylation, neuronal identity, connectivity, and function (99). While accumulating evidence implicates epigenetic dysregulation in the pathophysiology of neurodevelopmental and neuropsychiatric disorders, the specific contribution of DNMT1-dependent mechanisms remains incompletely understood. Existing findings suggest that altered DNMT1 expression, localization or recruitment may influence transcriptional programs relevant for synaptic function, inhibitory neurotransmission, and neuronal plasticity (21, 100, 101). However, in many cases, direct causal links between DNMT1 dysfunction and disease-associated phenotypes have yet to be established, highlighting an important area for future investigation.

### 5.2 DNMT1 in schizophrenia: epigenetic dysregulation of inhibitory circuits

The strongest evidence for DNMT1 involvement in neuropsychiatric disease comes from schizophrenia. Postmortem studies in individuals with schizophrenia have reported increased DNMT1 expression in cortical GABAergic interneurons (22). This increase has been linked to reduced expression of inhibitory genes, especially *RELN*, *GAD1* (*GAD67*) and *BDNF*, which are important for cortical circuit maturation and function (102–104). By contributing to promoter hypermethylation and transcriptional repression, DNMT1 may impair inhibitory neurotransmission and disrupt cortical excitation-inhibition balance (19). Among cortical inhibitory interneurons, parvalbumin-positive (PV^+^) interneurons represent a major fast-spiking subtype critically involved in maintaining this balance and coordinating cortical network activity (105). In adult mice, DNMT1 has been implicated in the epigenetic regulation of PV^+^ interneuron function, as interneuron-specific *Dnmt1* deletion altered inhibitory signaling and increased extracellular GABA levels without changes in interneuron number (28). These functional changes were accompanied by hypomethylation and upregulation of genes involved in clathrin-mediated endocytosis, suggesting that DNMT1-dependent DNA methylation modulates synaptic vesicle recycling and GABAergic transmission in PV^+^ interneurons (28). Given the central role of PV^+^ interneurons in maintaining cortical excitation-inhibition balance, DNMT1-dependent alterations in their inhibitory function may contribute to circuit dysfunction associated with schizophrenia (106, 107). These findings support the idea that epigenetic dysregulation of GABAergic interneurons contributes to cognitive deficits and circuit dysfunction in schizophrenia.

### 5.3 DNMT1 in autism spectrum disorders: emerging and heterogeneous evidence

In autism spectrum disorder (ASD), evidence for DNMT1 involvement remains limited and heterogeneous. Although ASD is associated with widespread alterations in DNA methylation, particularly in GABAergic circuits, the specific contribution of DNMT1 is still unclear (101, 108). Genetic studies suggest that variations in DNA methyltransferase genes, including *Dnmt1*, may influence methylation patterns associated with ASD predisposition (109). Postmortem studies provide suggestive but region-specific findings: in the cerebellum, increased methyl-CpG-binding protein 2 (MeCP2) binding, increased ten-eleven translocation methylcytosine dioxygenase 1 (TET1) expression, and 5-hydroxymethylcytosine (5hmC) enrichment at the *RELN* and *GAD1* promoters were found while *DNMT1* mRNA levels remained unchanged (110). In contrast, a later study in the frontal cortex found reduced *RELN* and *GAD1* mRNA expression together with increased DNMT1 and MeCP2 binding at *RELN*, *GAD1*, and *GAD2* promoters without significant changes in *DNMT1* mRNA levels (111). The current literature supports DNMT1 as a plausible modulator of ASD-associated epigenetic dysregulation, but not yet as a consistently established or central mechanism (112).

### 5.4 DNMT1 in depression: stress-associated epigenetic changes

The role of DNMT1 in depression has primarily been investigated in the context of stress-induced epigenetic changes (113, 114). Experimental studies indicate that stress can alter DNA methylation in brain regions involved in emotional regulation, such as the hippocampus or the amygdala, and that pharmacological or genetic modulation of DNMT1 levels can influence depression- and anxiety-like behaviors in animal models (43, 115–118). Increasing evidence suggests that these stress-related effects occur on a background of developmentally programmed vulnerability: early-life experiences, particularly during sensitive periods of brain development, can induce long-lasting epigenetic modifications that shape stress responsivity and emotional behavior later in life (113, 119). In this framework, DNMT1-dependent methylation during early development may contribute to the establishment of early epigenetic programs that persist into adulthood and influence later susceptibility to depression (115).

In humans, evidence supports a broader role for epigenetic dysregulation in major depressive disorder, although findings are often region- and cell type-specific (114, 115). While sex-specific differences in epigenetic regulation have been proposed, robust evidence for DNMT1-specific effects in this context remains limited (115).

### 5.5 Converging mechanisms and limitations

Across neurodevelopmental and neuropsychiatric disorders, DNMT1-related alterations converge on genes tied to inhibitory neurotransmission, synaptic organization, and neuronal plasticity. Dysregulation of DNMT1 can alter the expression of key genes such as *RELN*, *GAD1*, and *BDNF*, which affects neuronal development and consequently proper long-term brain function (102, 120). However, DNMT1 operates within a broader epigenetic network of DNMTs, TET enzymes, methyl-CpG binding proteins, and chromatin modifiers (101, 102, 110). Disease-associated epigenetic changes likely arise from coordinated disruptions across multiple regulatory systems rather than from DNMT1 alone. The causal versus correlative status of DNMT1 dysregulation remains difficult to resolve in human postmortem and clinical studies.

## 6 DNMT1 in neurodegenerative diseases

### 6.1 DNMT1 mutations define a spectrum of neurodegenerative disorders

Pathogenic *DNMT1* mutations cause a spectrum of adult-onset neurodegenerative disorders, most prominently hereditary sensory and autonomic neuropathy with dementia and hearing loss (HSAN1E), and autosomal dominant cerebellar ataxia, deafness and narcolepsy (ADCA-DN) (16). *DNMT1* mutations within exon 20 cause decreased enzymatic activity, DNMT1 mislocalization, and disrupted heterochromatin binding, resulting in global hypomethylation and local hypermethylation, which are thought to impair neuronal survival and function, and ultimately result in global brain atrophy (16, 18). Subsequent work revealed mutations in exon 21 of the *DNMT1* gene, extending the genetic association to ADCA-DN, now considered an allelic condition (23).

Disease-associated mutations in both syndromes cluster within the replication foci targeting sequence (RFTS) domain, a regulatory region that plays a critical role in controlling DNMT1 activity and its recruitment to chromatin (16, 121). Consistently, a mutation-induced proteolytic cleavage of DNMT1 in this domain has been shown to destabilize DNMT1 and induce neurodegenerative phenotypes in mice (122). Because of these mechanistic similarities and the substantial phenotypic overlap, the two conditions are now often considered part of a broader DNMT1-related neurodegenerative spectrum disorder (16, 23, 123).

While DNMT1 mutations lead to widespread epigenetic dysregulation, the resulting neurodegenerative phenotypes display a marked region- and cell-type-specific pattern. Selective neuronal vulnerability is a key feature of neurodegenerative diseases, whereby specific neuronal populations exhibit differential susceptibility to pathology. This vulnerability is thought to arise from intrinsic cellular properties, including differences in metabolic demand, axonal architecture, calcium handling, proteostatic capacity, and network connectivity (124, 125). Emerging evidence suggests that epigenetic mechanisms may contribute to the establishment and maintenance of these cell-type-specific transcriptional states underlying neuronal resilience and vulnerability. However, whether DNMT1-dependent DNA methylation programs directly contribute to selective neuronal vulnerability remains unclear and represents an important area for future research (125).

Recent advances in single-cell and spatial multi-omic approaches are beginning to resolve neuronal heterogeneity underlying selective vulnerability in neurodegenerative diseases (124). By enabling the parallel assessment of transcriptomic and epigenomic states at cellular resolution, these technologies may help determine whether epigenetic programs, including DNA methylation pathways regulated by DNMT1, contribute to differential susceptibility of neuronal populations. Direct evidence for a causal role of DNMT1 in selective neuronal vulnerability, however, remains limited.

### 6.2 Molecular mechanisms in DNMT1-related neurodegeneration: beyond maintenance methylation and non-canonical functions

Disease mechanisms in DNMT1-related neurodegeneration extend beyond altered methylation alone. Mutant DNMT1 proteins exhibit abnormal subcellular localization, reduced binding to heterochromatin, and a tendency to form cytoplasmic aggresomes, suggesting impaired protein folding and proteostasis (16). *DNMT1* mutations have also been associated with mitochondrial and metabolic dysfunction: in patient-derived cells, they were linked to reduced ATP levels, activation of AMP-activated protein kinase (AMPK) signaling, and inhibition of mammalian target of rapamycin complex 1 (mTORC1) pathways (50). Experimental studies further suggest that DNMT1 modulates proteostasis-relevant pathways such as retrograde trafficking and autophagy, thereby influencing the handling of aggregation-prone proteins and neuronal survival (126). Recent work also demonstrated a role for cytosolic DNMT1 in microtubule regulation and neuronal morphogenesis, further supporting the idea that DNMT1-related neurodegeneration involves mechanisms beyond altered DNA methylation (34). DNMT1 has also been implicated in DNA damage responses and genome stability, indicating broader cellular consequences of DNMT1 alterations (127, 128). Together, these findings point to a convergence of epigenetic dysregulation, proteostasis defects, metabolic dysfunction, and impaired genome maintenance in DNMT1-related neurodegeneration.

### 6.3 DNMT1 in Parkinson’s disease, Alzheimer’s disease and other neurodegenerative conditions

Beyond monogenic disorders, DNMT1 has also been implicated in more common neurodegenerative diseases. Parkinson’s disease (PD) is a progressive neurodegenerative disorder characterized by the degeneration of dopaminergic neurons in the substantia nigra and the presence of intracellular protein aggregates known as Lewy bodies, which are primarily composed of misfolded α-synuclein. These pathological features are shared with related synucleinopathies such as dementia with Lewy bodies [DLB; (129, 130)]. In PD, α-synuclein can sequester DNMT1 from the nucleus to the cytoplasm, resulting in reduced nuclear DNMT1 levels and global DNA hypomethylation in PD and DLB (17). This redistribution is associated with altered methylation at disease-relevant loci, including *SNCA*, suggesting a potential feed-forward mechanism in which α-synuclein accumulation further promotes its own expression through epigenetic dysregulation. DNMT1 variants have been associated with increased PD risk, and reduced DNMT1 levels have been reported in blood cells of PD patients, suggesting that DNMT1 dysregulation may contribute to PD susceptibility (131, 132).

In Alzheimer’s disease (AD), a progressive neurodegenerative disorder characterized by the accumulation of extracellular amyloid-β plaques and intracellular neurofibrillary tangles (NFTs) composed of hyperphosphorylated tau, widespread neuronal loss occurs particularly in the hippocampus and cortical regions (133). Alterations in DNA methylation and methylation-related factors have been reported, but evidence for DNMT1 involvement remains heterogeneous (134). Some studies reported increased methylation in the hippocampus, as well as in the middle frontal and middle temporal gyrus, which positively correlated with amyloid-β plaques, NFTs, and ubiquitin loads (135, 136), while others observed decrements in DNMT1-associated signals in neurons of the entorhinal cortex and the hippocampus (20, 137). Collectively, these heterogeneous findings point to a complex and region-dependent dysregulation of DNA methylation in AD. DNMT1-related changes in AD are better interpreted as part of a broader disruption of epigenetic homeostasis during disease progression rather than as a primary causal mechanism (134).

Emerging evidence links DNMT1 to amyotrophic lateral sclerosis (ALS), a progressive neurodegenerative disorder characterized by the degeneration of upper and lower motor neurons in the motor cortex, brainstem, and spinal cord (138). In addition to hallmark pathological features such as protein aggregation and neuronal loss, alterations in DNA methylation and DNA methyltransferase expression have been reported in motor neurons and spinal cord tissue from ALS patients. These observations suggest that epigenetic dysregulation may accompany disease progression and neuronal degeneration. Several reviews have subsequently highlighted epigenetic mechanisms, including DNA methylation changes, as potential contributors to ALS pathology (139, 140). Early studies reported increased DNMT1 expression and global DNA methylation changes in motor neurons from ALS patients, which were associated with apoptotic processes (141, 142). These alterations were accompanied by changes in other DNMTs, suggesting broader epigenetic remodeling in affected neurons (141). As in AD, a direct mechanistic role of DNMT1 in ALS pathogenesis has not been established, and DNMT1 dysregulation in ALS is more likely to be part of a broader epigenetic response associated with neuronal stress, degeneration, and altered gene expression (139, 140).

## 7 Discussion

**Figure 1:**
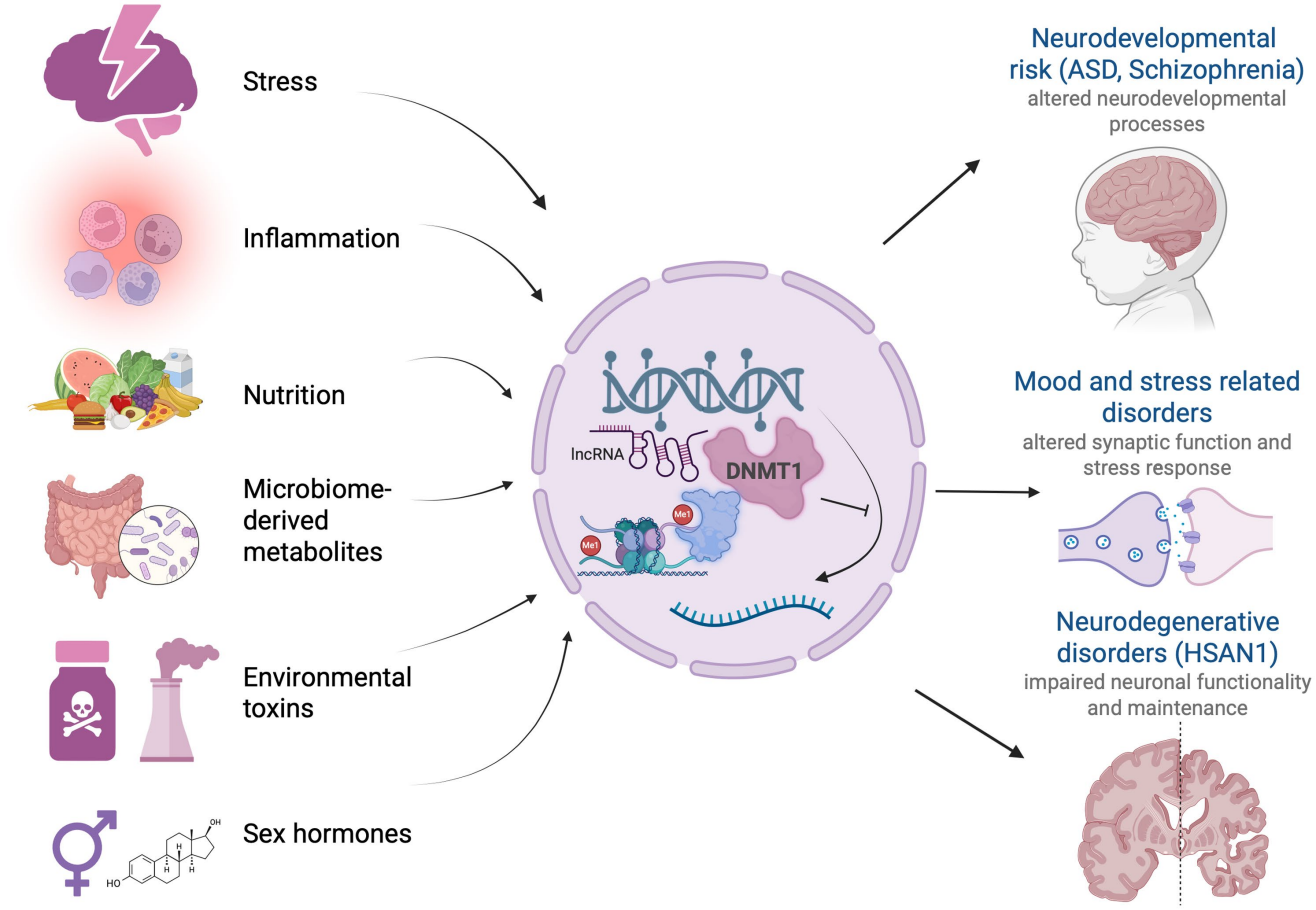
DNMT1 as an environmental epigenetic sensor. Schematic illustration of how diverse environmental and endogenous signals converge on DNMT1 to shape brain function and disease risk. Psychosocial stress, inflammation, nutrition, microbiome-derived metabolites, environmental toxicants and sex hormones modulate DNMT1 expression, localization and activity, in part via lncRNA-mediated targeting, thereby altering DNA methylation patterns in neurons and glia. These DNMT1-dependent methylation changes impact neurodevelopmental processes and circuit formation, contributing to neurodevelopmental risk (e.g., ASD, schizophrenia), influence synaptic plasticity and stress responsivity in mood and stress-related disorders, and impair neuronal maintenance and survival in DNMT1-associated neurodegenerative conditions such as HSAN1. Created in BioRender. Zimmer-Bensch, G. (144) https://BioRender.com/ima9w20.

The body of evidence reviewed here establishes DNMT1 as a key environmental sensor and epigenetic hub in the nervous system. By integrating signals from stress, nutrition, the microbiome, environmental toxicants, sex hormones, and non-coding RNAs, DNMT1 translates a diverse array of environmental inputs into long-lasting changes in DNA methylation landscapes, non-canonical histone crosstalk, and subcellular localization in neurons (Figure 1). This environmentally responsive capacity positions DNMT1 as a central mechanistic bridge between the epigenome and disease vulnerability across the lifespan.

A key conceptual advance is the recognition that environmental perturbation of DNMT1 engages a multilayered regulatory architecture: metabolite availability governs enzymatic capacity; PTMs link genotoxic, oxidative, and activity-dependent stimuli to DNMT1 stability and recruitment; lncRNAs provide locus specificity; and sex hormone signaling introduces sex-stratified dimensions that may explain observed sex differences in disease incidence and epigenetic responses to environmental exposures. The convergence of these mechanisms on shared target gene networks—particularly genes regulating inhibitory neurotransmission such as *RELN*, *GAD1*, and *BDNF*—provides a parsimonious explanation for why diverse environmental insults converge on similar neuropsychiatric phenotypes.

Important limitations temper the conclusions that can currently be drawn. A substantial proportion of mechanistic insights derives from rodent models and cancer cell lines, and translating these to the human brain requires caution. The causal versus correlative status of DNMT1 dysregulation in common neuropsychiatric and neurodegenerative diseases remains difficult to resolve. Furthermore, the preponderance of published studies has used male animals exclusively, leaving sex-specific mechanisms largely uncharacterized despite compelling evidence for ERα-DNMT1 feedback loops and sex-stratified responses to environmental stressors. Nearly all existing work also captures epigenetic states at single time points; longitudinal studies tracking DNMT1-dependent methylation trajectories from early development through aging are lacking.

Future advances in single-cell and spatial epigenomics, long-read sequencing, CRISPR-based epigenome editing, and human iPSC-derived neural organoid models will be essential for addressing these gaps. On the translational front, understanding how environmental exposures perturb DNMT1-centered pathways opens avenues for early biomarker development and potentially for targeted epigenetic interventions in at-risk individuals. DNMT inhibitors already approved for hematological malignancies could provide a pharmacological scaffold from which more brain-penetrant and potentially cell-type-selective compounds could be developed, provided that mechanistic understanding is sufficiently refined to anticipate on-target and off-target effects in the nervous system (10, 143). Taken together, DNMT1 emerges as one of the most instructive entry points into understanding how environmental experiences become biologically embedded in the epigenome and shape brain health across the lifespan.
